# The Role of the C‐Reactive Protein–Triglyceride Glucose Index in Predicting New‐Onset Chronic Diseases: Evidence From a Longitudinal Cohort Study

**DOI:** 10.1002/brb3.71299

**Published:** 2026-02-28

**Authors:** Huang Luwen, Mei Lijun, Li Linlin, Yu Ming

**Affiliations:** ^1^ Department of Neurology Suining Central Hospital Suining Sichuan China

**Keywords:** C‐reactive protein–triglyceride glucose index, chronic diseases, cohort study

## Abstract

**Background:**

The C‐reactive protein–triglyceride glucose index (CTI) is an emerging biomarker reflecting both systemic inflammation and insulin resistance. However, its association with the risk of new‐onset chronic diseases remains insufficiently studied.

**Methods:**

Data were derived from the China Health and Retirement Longitudinal Study between 2011 and 2020. A total of 9275 participants were included. This study assessed the associations between CTI levels and 14 chronic diseases, including hypertension, dyslipidemia, diabetes, stroke, liver disease, lung disease, osteoarthritis, and other diseases. Cox proportional hazards models were used to estimate the HRs for disease incidence, adjusting for confounders. Restricted cubic spline analyses were performed to explore potential nonlinear relationships.

**Results:**

Elevated CTI levels were significantly associated with increased risks of new‐onset hypertension (OR = 1.411, 95% CI: 1.274, 1.563), dyslipidemia (OR = 1.645, 95% CI: 1.508, 1.793), DM (OR = 1.932, 95% CI: 1.724, 2.165), stroke (OR = 1.676, 95% CI: 1.491, 1.883), and liver disease (OR = 1.279, 95% CI: 1.124, 1.455). A significant nonlinear association was observed between the CTI and osteoarthritis (*p*‐nonlinear = 0.03) as well as stroke (*p*‐nonlinear = 0.012).

**Conclusions:**

Elevated CTI is strongly associated with an increased risk of several chronic diseases, highlighting its potential value as a clinical risk assessment tool and predictive biomarker.

## Graphical abstract

Elevated CTI was significantly associated with increased risks of new‐onset hypertension, diabetes, dyslipidemia, stroke, liver disease, and osteoarthritis.

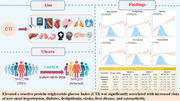

## 1 Introduction

Chronic diseases represent a significant and growing burden on individuals, families, and society, demanding increasingly specialized healthcare services and increasing medical costs (Nugent 2019). From 2010 to 2021, noncommunicable diseases accounted for the largest proportion of the global disease burden, being the sole group responsible for the increase in disability‐adjusted life years (GBD 2021 Diseases and Injuries Collaborators 2024). As the country with the largest elderly population, China faces significant challenges associated with aging, with the economic burden projected to reach $7.7 trillion between 2010 and 2030 (Bloom et al. 2020). Consequently, identifying modifiable risk factors and implementing effective preventive measures are essential for the primary prevention of chronic diseases.

The C‐reactive protein–triglyceride glucose index (CTI), a novel biomarker that integrates systemic inflammation and insulin resistance (IR), is composed of C‐reactive protein (CRP), triglyceride (TG), and fasting blood glucose (FBG) levels and has attracted increasing attention in recent years (Ren et al. 2025; Huo et al. 2025; Mei et al. 2024). Compared with the triglyceride–glucose (TyG) index, the CTI combines these three markers and has demonstrated superior efficacy in predicting the risk of stroke, coronary heart disease, and Type 2 diabetes over traditional individual biomarkers (Huo et al. 2025; Tang et al. 2025; Xu et al. 2024). However, the potential associations between CTI and various other diseases, particularly the onset of chronic conditions, remain inadequately explored. Most existing studies on CTI and chronic diseases are based on cross‐sectional data, highlighting the need for large‐scale longitudinal research. Moreover, the coexistence of multiple chronic diseases may have synergistic effects. Therefore, a systematic evaluation of the ability of the CTI to predict different chronic diseases is essential.

To address these critical research gaps, data from the China Health and Retirement Longitudinal Study (CHARLS) were analyzed to explore the associations between CTI and chronic diseases. The aim of this study was to determine whether the CTI can be established as a novel biomarker for the risk stratification of chronic diseases and to provide insights for targeted preventive strategies.

## 2 Methods

### 2.1 Study Design and Population

Data for this analysis were obtained from the CHARLS, a prospective cohort study of individuals in China (Zhao et al. 2014). The participants were selected from 450 communities across 150 county‐level units in 28 provinces. The study collected detailed sociodemographic, health, and laboratory data through surveys conducted every 2 years. The baseline survey was conducted in 2011, followed by follow‐up surveys in 2013, 2015, 2018, and 2020 (Waves 2, 3, 4, and 5).

**FIGURE 1:**
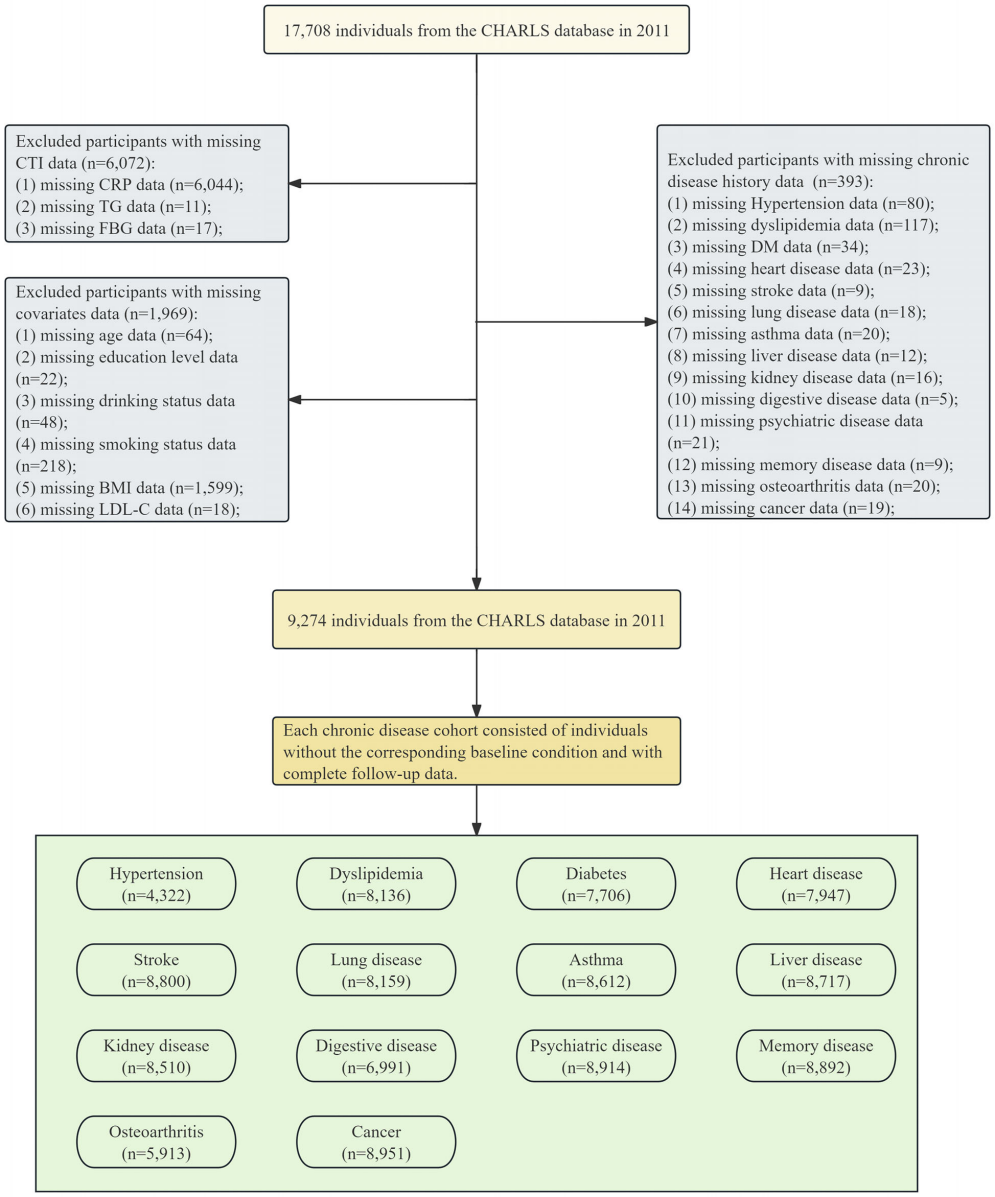
Flowchart of the study population.

A total of 17,708 participants were included in 2011. Participants were excluded on the basis of the following criteria: missing CTI data (*n* = 6072), incomplete chronic disease information (*n* = 393), and missing covariate data (*n* = 64). Each chronic disease cohort was composed of individuals without the corresponding disease at baseline and with complete follow‐up data. A detailed flowchart of the selection process is shown in Figure 1.

The CHARLS study was approved by the Biomedical Ethics Review Board of Peking University (IRB00001052–11015), and written informed consent was obtained from all participants prior to their involvement (Zhao et al. 2014).

### 2.2 CTI Calculation

The CTI was calculated via the following formula:

$$\begin{array}{c} \mathrm{CTI}=0.412\times \ln\left(\mathrm{CRP}\left[\mathrm{mg}/L\right]\right)+\ln\left(\mathrm{TG}\left[\mathrm{mg}/\mathrm{dL}\right]\times \mathrm{FBG}\left[\mathrm{mg}/\mathrm{dL}\right]\right)\\ /2\;(\text{Ruan et al}.2022). \end{array}$$

### 2.3 Outcome Definition

For each chronic disease cohort, incident cases were identified when participants answered “yes” to the question “Has a doctor ever diagnosed you with [a specific disease]?” during follow‐up assessments (Zhao et al. 2014; Zhuo et al. 2024). For example, the incidence of hypertension was based on self‐reported physician diagnoses from any follow‐up wave (2013–2020). Participants who reported a physician's diagnosis of hypertension were classified as having hypertension. The time to hypertension onset was calculated from baseline to the first reported diagnosis.

### 2.4 Covariates

The analysis included demographic factors such as age, sex, marital status, residence, and education level; lifestyle factors such as drinking and smoking status; and BMI. The laboratory parameters considered in the analysis included CRP, FBG, total cholesterol (TC), TG, low‐density lipoprotein cholesterol (LDL‐C), high‐density lipoprotein cholesterol (HDL‐C), blood urea nitrogen (BUN), and uric acid. The analysis also included 14 chronic diseases as covariates: hypertension, dyslipidemia, diabetes, heart disease, stroke, lung disease, asthma, liver disease, kidney disease, digestive disease, psychiatric disease, memory disease, osteoarthritis, and cancer. Each disease cohort excluded the specific disease being investigated.

### 2.5 Statistical Analysis

The participants were categorized into four groups on the basis of CTI quartiles, and the CTI was also analyzed as a continuous variable to increase the robustness of the results. The risk of chronic diseases was assessed using a competing risk survival model and Gray's test to account for the possibility that multiple events may prevent the occurrence of other events. Specifically, we applied the Fine and Gray model, which estimates the cumulative incidence function for each disease while considering the presence of competing risks. Associations between the CTI score and new‐onset chronic diseases were evaluated via logistic regression models. Three models were developed: Model 1, which was unadjusted; Model 2, which was adjusted for sex, age, residence, marital status, education level, smoking status, drinking status, and BMI; and Model 3, which was further adjusted for LDL‐C and 14 chronic disease histories, excluding the specific disease under investigation in each cohort. A fully adjusted restricted cubic spline (RCS) analysis was conducted to investigate the dose–response relationship, and threshold effects were analyzed to identify the inflection point. Subgroup analyses were performed on the basis of factors such as age, sex, education level, marital status, and BMI.

To validate the findings, two sensitivity analyses were conducted. First, Cox regression was applied to clarify the relationship between the CTI and the risk of chronic diseases. Furthermore, participants who experienced outcome events during Wave 2 were excluded to clarify the relationship between the CTI and the risk of chronic diseases.

All analyses were conducted via R version 4.2.1 (http://www.R‐project.org) and Free Statistics version 2.1.1. A *p* value of less than 0.05 was considered statistically significant.

## 3 Results

### 3.1 Baseline Characteristics

Table 1 presents the baseline characteristics of the 9274 participants stratified by CTI quartile. Higher CTI quartiles were associated with older age, poorer self‐comment of health, higher prevalence of smoking and alcohol consumption, increased BMI, and adverse laboratory parameters, including higher CRP, FBG, TC, TG, and LDL‐C levels and lower HDL‐C levels (all *p* < 0.001) (Table S1). The prevalence of chronic diseases such as hypertension, diabetes, dyslipidemia, and stroke also increased with CTI (all *p* < 0.001). To evaluate potential selection bias due to missing CTI data, we compared baseline characteristics between participants with CTI data (*n* = 11,636) and those excluded due to missing CTI (*n* = 6072) (Table S2). The excluded group was slightly younger, had a higher proportion of females and urban residents, and differed in marital status and education level (all *p* < 0.001), and also showed differences in BMI, TC, and HDL‐C (*p* < 0.05). In addition, the excluded group had a lower prevalence of hypertension, dyslipidemia, and diabetes but higher prevalence of kidney disease, digestive disease, and osteoarthritis (*p* < 0.05). By contrast, smoking status, alcohol consumption, heart disease, stroke, lung disease, asthma, liver disease, psychiatric disease, and cancer did not differ significantly between groups (all *p* > 0.05). These findings suggest that, despite some differences, many key clinical characteristics were similar between included and excluded participants, supporting the representativeness of the analytic sample.

**TABLE 1:** Baseline characteristics of the CHARLS participants by CTI index quartile.

Variables	Total	Q1 (< 4.33)	Q2 (4.33–4.71)	Q3 (4.72–5.12)	Q4 (> 5.12)	*p* value
*N*	9275	2319	2318	2319	2319	
Age, years	59.1 ± 9.7	57.3 ± 9.4	59.1 ± 9.7	59.7 ± 9.8	60.3 ± 9.5	< 0.001
Sex (female), *n* (%)	5033 (54.3)	1271 (54.8)	1211 (52.2)	1276 (55)	1275 (55)	0.164
Marital status (married), *n* (%)	8146 (87.8)	2069 (89.2)	2038 (87.9)	2035 (87.8)	2004 (86.4)	0.036
Residence (urban), *n* (%)	3255 (35.1)	701 (30.2)	752 (32.4)	872 (37.6)	930 (40.1)	< 0.001
Education level, *n* (%)						0.44
Elementary school or below	6501 (70.1)	1609 (69.4)	1654 (71.4)	1612 (69.5)	1626 (70.1)	
Middle school	1870 (20.2)	480 (20.7)	439 (18.9)	494 (21.3)	457 (19.7)	
High school or above	904 (9.7)	230 (9.9)	225 (9.7)	213 (9.2)	236 (10.2)	
Drinking status, *n* (%)						< 0.001
Never	5505 (59.4)	1351 (58.3)	1342 (57.9)	1390 (59.9)	1422 (61.3)	
Former	775 (8.4)	165 (7.1)	188 (8.1)	216 (9.3)	206 (8.9)	
Now	2995 (32.3)	803 (34.6)	788 (34)	713 (30.7)	691 (29.8)	
Smoking status, *n* (%)						< 0.001
Never	5694 (61.4)	1463 (63.1)	1397 (60.3)	1425 (61.4)	1409 (60.8)	
Former	816 (8.8)	156 (6.7)	191 (8.2)	224 (9.7)	245 (10.6)	
Now	2765 (29.8)	700 (30.2)	730 (31.5)	670 (28.9)	665 (28.7)	
BMI (kg/m2)	23.2 (20.9, 25.8)	21.9 (20.1, 23.9)	22.6 (20.5, 24.9)	23.9 (21.6, 26.5)	24.7 (22.0, 27.6)	< 0.001
Laboratory parameters						
CRP (mg/L)	1.0 (0.6, 2.2)	0.4 (0.3, 0.6)	0.8 (0.6, 1.1)	1.5 (1.0, 2.2)	3.5 (1.9, 7.1)	< 0.001
FBG (mg/L)	110.1 ± 36.9	97.5 ± 15.9	103.3 ± 20.1	108.9 ± 25.3	130.9 ± 59.3	< 0.001
TC (mg/dl)	193.43 ± 38.27	183.46 ± 33.27	191.97 ± 36.82	195.50 ± 36.93	202.80 ± 42.88	< 0.001
TG (mg/dl)	105.32 (75.22, 153.99)	71.68 (56.64, 90.27)	99.12 (76.11, 130.10)	123.01 (92.04, 162.84)	169.92 (114.17, 55.32)	< 0.001
LDL‐C (mg/dl)	116.48 ± 35.03	111.05 ± 29.11	118.86 ± 33.10	120.37 ± 35.13	115.63 ± 41.01	< 0.001
HDL‐C (mg/dl)	51.20 ± 15.24	59.30 ± 14.85	53.60 ± 14.07	48.99 ± 13.59	42.91 ± 13.46	< 0.001
BUN (mg/dl)	15.72 ± 4.60	15.87 ± 4.71	15.86 ± 4.59	15.63 ± 4.45	15.50 ± 4.62	0.013
UA (mg/dl)	4.45 ± 1.26	4.08 ± 1.11	4.33 ± 1.18	4.57 ± 1.23	4.81 ± 1.37	< 0.001
CTI	4.76 ± 0.59	4.06 ± 0.21	4.52 ± 0.11	4.90 ± 0.12	5.55 ± 0.37	< 0.001
Chronic diseases						
Hypertension, *n* (%)	4431 (47.77)	824 (35.53)	1008 (43.49)	1232 (53.13)	1367 (58.95)	< 0.001
Dyslipidemia, *n* (%)	918 (9.90)	125 (5.39)	182 (7.85)	250 (10.78)	361 (15.57)	< 0.001
Diabetes, *n* (%)	1373 (14.80)	126 (5.43)	209 (9.02)	332 (14.32)	706 (30.44)	< 0.001
Heart disease, *n* (%)	1110 (11.97)	200 (8.62)	249 (10.74)	294 (12.68)	367 (15.83)	< 0.001
Stroke, *n* (%)	230 (2.48)	34 (1.47)	56 (2.42)	57 (2.46)	83 (3.58)	< 0.001
Lung disease, *n* (%)	908 (9.79)	205 (8.84)	231 (9.97)	217 (9.36)	255 (11)	0.08
Asthma, *n* (%)	426 (4.59)	98 (4.23)	91 (3.93)	105 (4.53)	132 (5.69)	0.024
Liver disease, *n* (%)	314 (3.39)	87 (3.75)	68 (2.93)	83 (3.58)	76 (3.28)	0.432
Kidney disease, *n* (%)	536 (5.78)	135 (5.82)	138 (5.95)	126 (5.43)	137 (5.91)	0.869
Digestive disease, *n* (%)	2101 (22.65)	611 (26.35)	520 (22.43)	495 (21.35)	475 (20.48)	< 0.001
Psychiatric disease, *n* (%)	116 (1.25)	39 (1.68)	22 (0.95)	33 (1.42)	22 (0.95)	0.058
Memory disease, *n* (%)	138 (1.49)	32 (1.38)	37 (1.6)	28 (1.21)	41 (1.77)	0.414
Osteoarthritis, *n* (%)	3192 (34.42)	742 (32)	817 (35.25)	796 (34.33)	837 (36.09)	0.022
Cancer, *n* (%)	78 (0.84)	11 (0.47)	21 (0.91)	19 (0.82)	27 (1.16)	0.079

Abbreviations: BMI, body mass index; BUN, blood urea nitrogen; CRP, C‐reactive protein; FBG, fasting blood glucose; HDL‐C, high‐density lipoprotein cholesterol; LDL‐C, low‐density lipoprotein cholesterol; TC, total cholesterol; TG, triglycerides; UA, uric acid.

### 3.2 Associations Between the CTI and New‐Onset Chronic Diseases

**FIGURE 2:**
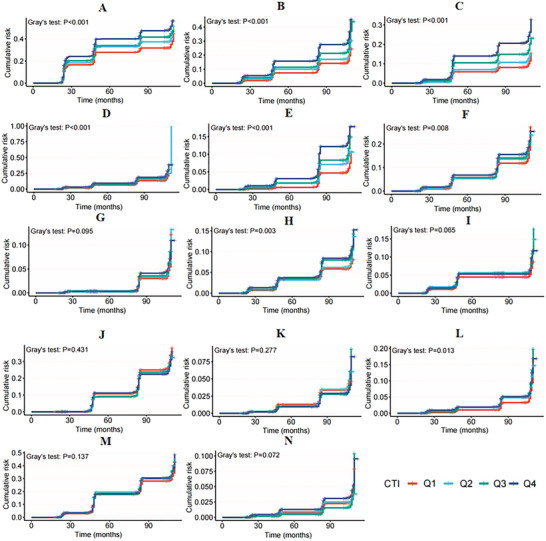
Competing risk model analysis depicting the cumulative incidence of chronic diseases across the CTI quartiles. (A) Hypertension, (B) dyslipidemia, (C) diabetes, (D) heart disease, (E) stroke, (F) lung disease, (G) asthma, (H) liver disease, (I) kidney disease, (J) digestive disease, (K) psychiatric disease, (L) memory disease, (M) osteoarthritis, and (N) cancer.

The competing risk survival model revealed that the cumulative incidence of HBP, dyslipidemia, DM, heart disease, and stroke increased with increasing CTI quartile, with participants in the highest quartile (Q4) showing the highest cumulative incidence of these conditions (Figure 2). Elevated CTI levels were significantly associated with increased risks of new‐onset hypertension (OR = 1.411, 95% CI: 1.274, 1.563), dyslipidemia (OR = 1.645, 95% CI: 1.508, 1.793), DM (OR = 1.932, 95% CI: 1.724, 2.165), stroke (OR = 1.676, 95% CI: 1.491, 1.883), and liver disease (OR = 1.279, 95% CI: 1.124, 1.455) (Table 2). These associations remained statistically significant after full adjustment. Additionally, in the fully adjusted model, elevated CTI values were also associated with an increased risk of new‐onset osteoarthritis (OR = 1.11, 95% CI: 1.003, 1.23). To further investigate this relationship, we explored the association between the CTI and new‐onset chronic diseases by stratifying participants into CTI quartiles (Table S3). The quartile‐based analysis revealed that compared with participants in the lowest quartile, those in the highest quartile (Q4) had significantly greater risks of new‐onset hypertension (OR = 1.596, 95% CI: 1.325, 1.923), dyslipidemia (OR = 1.744, 95% CI: 1.472, 2.067), diabetes (OR = 2.425, 95% CI: 1.472, 2.067), stroke (OR = 2.053, 95% CI: 1.59, 2.651), lung disease (OR = 1.278, 95% CI: 1.041, 1.569), liver disease (OR = 1.307, 95% CI: 1.008, 1.658), and osteoarthritis (OR = 1.186, 95% CI: 1.008, 1.658), with all trends showing statistical significance (all *p* < 0.05). However, the association with heart disease was weaker, and no significant associations were found for kidney disease, digestive diseases, psychiatric disorders, or cancer.

**TABLE 2:** Logistic regression analysis of the association between CTI and new‐onset chronic diseases in CHARLS participants.

Variables	Event, (*n*%)	Model 1		Model 2		Model 3	
		OR (95% CI)	*p* value	OR (95% CI)	*p* value	OR (95% CI)	*p* value
Hypertension	1962 (40.5)	1.411 (1.274, 1.563)	< 0.001	1.31 (1.177, 1.458)	< 0.001	1.286 (1.152, 1.437)	< 0.001
Dyslipidemia	2009 (24)	1.645 (1.508, 1.793)	< 0.001	1.661 (1.521, 1.814)	< 0.001	1.5 (1.364, 1.649)	< 0.001
Diabetes	1122 (14.2)	1.932 (1.724, 2.165)	< 0.001	1.933 (1.722, 2.169)	< 0.001	1.788 (1.587, 2.014)	< 0.001
Heart disease	1509 (18.5)	1.221 (1.111, 1.343)	< 0.001	1.158 (1.051, 1.276)	0.003	1.04 (0.936, 1.156)	0.463
Stroke	818 (9)	1.676 (1.491, 1.883)	< 0.001	1.613 (1.432, 1.816)	< 0.001	1.418 (1.245, 1.615)	< 0.001
Lung disease	1254 (15)	1.109 (1.002, 1.228)	0.046	1.098 (0.989, 1.218)	0.080	1.109 (0.99, 1.242)	0.075
Asthma	408 (4.6)	1.181 (1.001, 1.395)	0.049	1.153 (0.973, 1.366)	0.101	1.119 (0.931, 1.345)	0.230
Liver disease	681 (7.6)	1.279 (1.124, 1.455)	< 0.001	1.288 (1.13, 1.469)	< 0.001	1.209 (1.049, 1.393)	0.009
Kidney disease	629 (7.2)	1.148 (1.002, 1.315)	0.047	1.131 (0.985, 1.3)	0.082	1.046 (0.901, 1.215)	0.556
Digestive disease	1784 (24.9)	0.922 (0.84, 1.011)	0.083	0.945 (0.859, 1.039)	0.243	0.937 (0.847, 1.036)	0.203
Psychiatric disease	330 (3.6)	0.885 (0.732, 1.071)	0.209	0.888 (0.726, 1.085)	0.246	0.82 (0.663, 1.015)	0.068
Memory disease	664 (7.3)	1.192 (1.044, 1.36)	0.009	1.11 (0.969, 1.272)	0.134	1.01 (0.873, 1.169)	0.890
Osteoarthritis	1874 (30.8)	1.028 (0.936, 1.128)	0.566	1.053 (0.957, 1.159)	0.288	1.11 (1.003, 1.23)	0.045
Cancer	280 (3)	1.117 (0.915, 1.363)	0.278	1.111 (0.907, 1.359)	0.309	1.067 (0.861, 1.322)	0.556

*Note*: Model 1: Crude model. Model 2: Adjusted for age, sex, education level, drinking status, smoking status, and BMI. Model 3: Adjusted for age, sex, education level, drinking status, smoking status, BMI, LDL‐C, and a history of 14 chronic diseases at baseline (excluding the chronic disease being studied in each cohort). In the dyslipidemia, TC was additionally included. In the kidney disease, UA, creatinine, and BUN were further adjusted.

**FIGURE 3:**
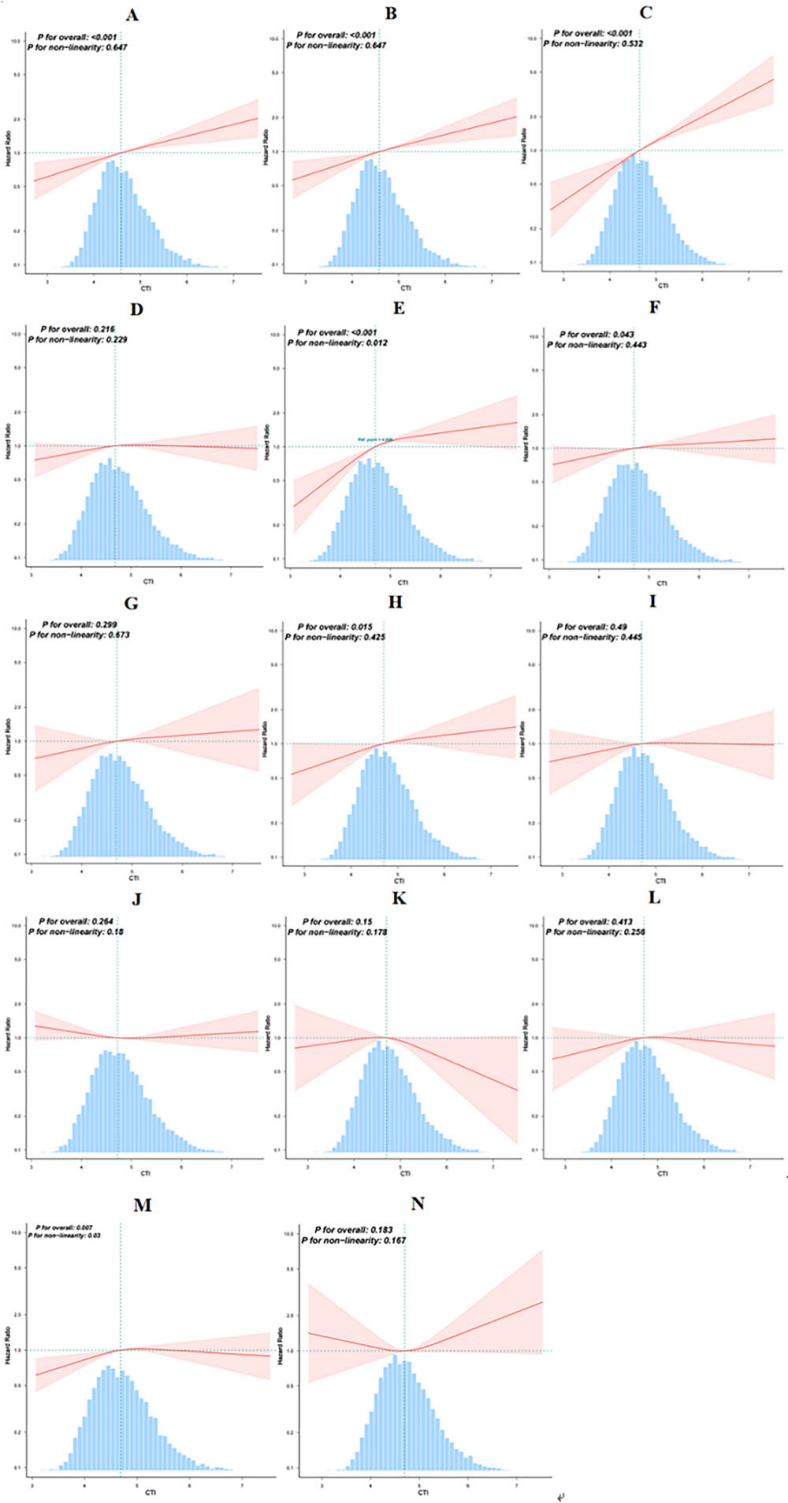
Association between the CTI and the risk of new‐onset chronic diseases. (A) Hypertension, (B) dyslipidemia, (C) diabetes, (D) heart disease, (E) stroke, (F) lung disease, (G) asthma, (H) liver disease, (I) kidney disease, (J) digestive disease, (K) psychiatric disease, (L) memory disease, (M) osteoarthritis, and (N) cancer. The model was adjusted for age, sex, education level, drinking status, smoking status, BMI, LDL‐C, and a history of 14 chronic diseases at baseline (excluding the chronic disease being studied in each cohort).

RCS analysis revealed a significant nonlinear relationship between CTI and the incidence of stroke (*p* for nonlinearity = 0.012) (Figure 3). Similarly, a significant nonlinear relationship was also found between CTI and the incidence of osteoarthritis (*p* for nonlinearity = 0.03) (Figure 3). Threshold analysis further revealed turning points at CTI = 5.173 for stroke (Table 3) and at CTI = 4.311 for osteoarthritis (Table 4). Below these thresholds, higher CTI values significantly increased the risk of stroke (HR = 1.709, 95% CI: 1.349, 2.165) and osteoarthritis (HR = 2.209, 95% CI: 1.32, 3.694). However, above *p* value for LRT test these thresholds, the association plateaued and became nonsignificant. In contrast, linear associations were observed between the CTI and diabetes, dyslipidemia, hypertension, liver disease, and lung disease (*p* for linear < 0.05).

**TABLE 3:** Threshold effect analysis of the relationship between CTI and the risk of stroke.

**Models**	**Per‐1 unit increase** **HR (95% CI)**	***p* value**
Model I	1.444 (1.279, 1.629)	< 0.001
One line effect		
Model II		
Turning point (K)	5.173 (5.085, 5.261)	
VAII < K	1.709 (1.349, 2.165)	< 0.001
VAII ≥ K	0.829 (0.55, 1.25)	0.371
*p* value for LRT test*		0.006

*Note*: Model I, linear analysis; Model II, nonlinear analysis. Adjusted for age, sex, education level, drinking status, smoking status, BMI, LDL‐C, and a history of 14 chronic diseases at baseline (excluding the chronic disease being studied in each cohort).

Abbreviation: LRT, logarithm likelihood ratio test.

*p <  0.05 indicates that Model II is significantly different from Model I.

**TABLE 4:** Threshold effect analysis of the relationship between CTI and the risk of osteoarthritis.

**Models**	**Per‐1 unit increase** **HR (95% CI)**	***p* value**
Model I	1.112 (1.017, 1.215)	0.019
One line effect		
Model II		
Turning point (K)	4.311 (4.28, 4.342)	
VAII < K	2.209 (1.32, 3.694)	0.003
VAII ≥ K	1.014 (0.898, 1.144)	0.826
*p* value for LRT test*		0.028

*Note*: Model I, linear analysis; Model II, nonlinear analysis. Adjusted for age, sex, education level, drinking status, smoking status, BMI, LDL‐C, and a history of 14 chronic diseases at baseline (excluding the chronic disease being studied in each cohort).

Abbreviation: LRT, logarithm likelihood ratio test.

*p <  0.05 indicates that Model II is significantly different from Model I.

### 3.3 Subgroup Analyses

Subgroup analyses were conducted to assess potential effect modifications by age, sex, education level, marital status, residential status, and BMI. The association between the CTI and the risk of new‐onset chronic diseases was consistent across subgroups, with no significant interactions observed (Figure 4) (all *p* values for interactions > 0.05), except for the interactions between age and dyslipidemia, sex and DM, and sex and digestive diseases.

FIGURE 4Subgroup analyses of the associations between the CTI and the risk of new‐onset chronic diseases. (A) Hypertension, (B) dyslipidemia, (C) diabetes, (D) heart disease, (E) stroke, (F) lung disease, (G) asthma, (H) liver disease, (I) kidney disease, (J) digestive disease, (K) psychiatric disease, (L) memory disease, (M) osteoarthritis, and (N) cancer. The model was adjusted for age, sex, education level, drinking status, smoking status, BMI, LDL‐C, and a history of 14 chronic diseases at baseline (excluding the chronic disease being studied in each cohort).

**Figure:**
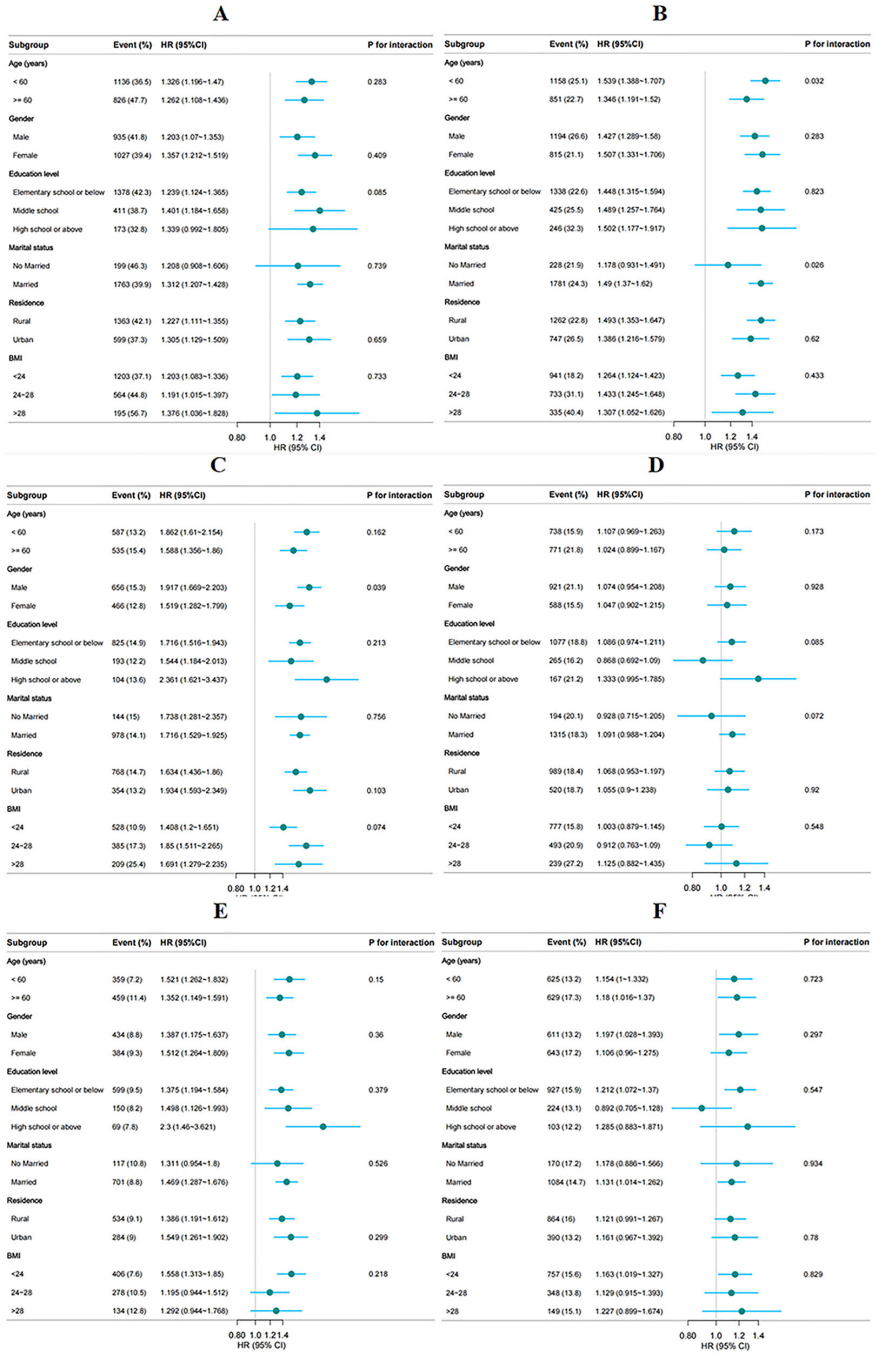

**Figure:**
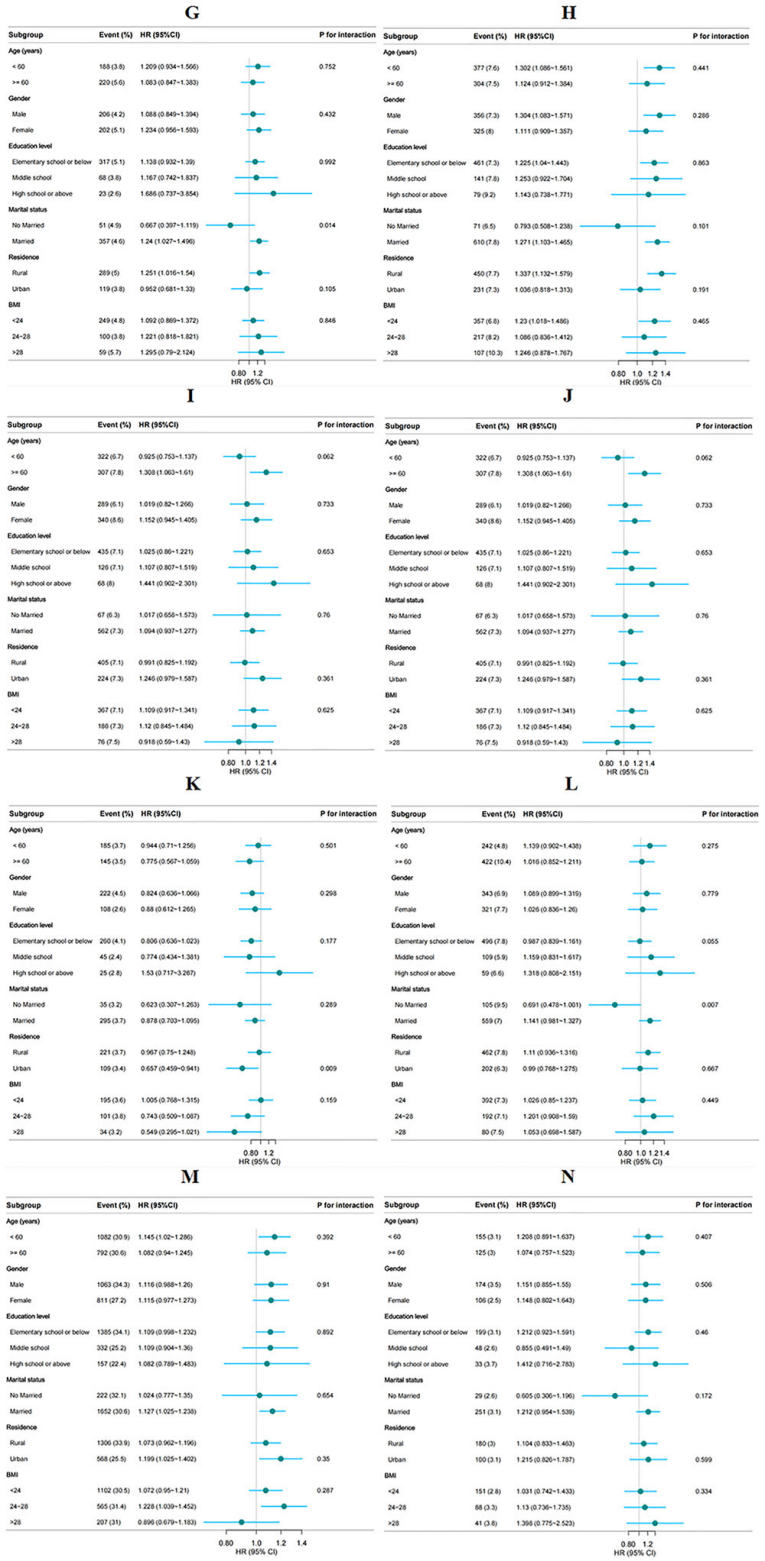

### 3.4 Sensitivity Analyses

First, Cox regression analysis was conducted to assess the robustness of the results (Table 5). The results from Table S4 also confirm the stability of the association between CTI quartiles and new‐onset chronic diseases through Cox regression analysis. Furthermore, after excluding participants who experienced outcome events during the first follow‐up (Wave 2), a subsequent analysis confirmed the robustness of these findings (Table S5).

**TABLE 5:** Cox regression analysis of the association between CTI and new‐onset chronic diseases in CHARLS participants.

Variables	Event, (*n*%)	Model 1		Model 2		Model 3	
		HR (95% CI)	*p* value	HR (95% CI)	*p* value	HR (95% CI)	*p* value
Hypertension	1962 (40.5)	1.364 (1.264, 1.472)	< 0.001	1.331 (1.232, 1.438)	< 0.001	1.305 (1.205, 1.414)	< 0.001
Dyslipidemia	2009 (24)	1.633 (1.52, 1.755)	< 0.001	1.614 (1.501, 1.737)	< 0.001	1.452 (1.342, 1.571)	< 0.001
Diabetes	1122 (14.2)	1.9 (1.716, 2.102)	< 0.001	1.878 (1.694, 2.081)	< 0.001	1.729 (1.554, 1.924)	< 0.001
Heart disease	1509 (18.5)	1.248 (1.147, 1.358)	< 0.001	1.18 (1.082, 1.286)	< 0.001	1.063 (0.969, 1.166)	0.195
Stroke	818 (9)	1.724 (1.546, 1.922)	< 0.001	1.648 (1.475, 1.841)	< 0.001	1.444 (1.279, 1.629)	< 0.001
Lung disease	1254 (15)	1.148 (1.045, 1.26)	0.004	1.123 (1.021, 1.236)	0.017	1.133 (1.024, 1.254)	0.016
Asthma	408 (4.6)	1.24 (1.055, 1.458)	0.009	1.2 (1.017, 1.415)	0.031	1.146 (0.961, 1.366)	0.130
Liver disease	681 (7.6)	1.325 (1.17, 1.5)	< 0.001	1.313 (1.158, 1.49)	< 0.001	1.215 (1.061, 1.391)	0.005
Kidney disease	629 (7.2)	1.191 (1.044, 1.358)	0.009	1.168 (1.02, 1.337)	0.024	1.077 (0.93, 1.246)	0.321
Digestive disease	1784 (24.9)	0.976 (0.901, 1.057)	0.550	0.99 (0.911, 1.077)	0.822	0.974 (0.892, 1.063)	0.549
Psychiatric disease	330 (3.6)	0.932 (0.773, 1.125)	0.465	0.937 (0.767, 1.144)	0.522	0.853 (0.692, 1.052)	0.137
Memory disease	664 (7.3)	1.252 (1.103, 1.421)	< 0.001	1.172 (1.029, 1.334)	0.017	1.051 (0.915, 1.208)	0.480
Osteoarthritis	1874 (30.8)	1.071 (0.992, 1.157)	0.080	1.077 (0.996, 1.164)	0.063	1.112 (1.017, 1.215)	0.019
Cancer	280 (3)	1.176 (0.965, 1.432)	0.108	1.154 (0.945, 1.41)	0.160	1.143 (0.912, 1.433)	0.246

*Note*: Model 1, crude model. Model 2, adjusted for age, sex, education level, drinking status, smoking status, and BMI. Model 3, adjusted for age, sex, education level, drinking status, smoking status, BMI, LDL‐C, and a history of 14 chronic diseases at baseline (excluding the chronic disease being studied in each cohort). In the dyslipidemia, TC was additionally included. In the kidney disease, UA, creatinine, and BUN were further adjusted.

## 4 Discussion

This study explored the association between the CTI and the risk of chronic diseases. These findings suggest that higher CTI levels are associated with an increased risk of developing hypertension, dyslipidemia, diabetes, stroke, liver disease, and osteoarthritis. According to the fully adjusted models, participants in the highest CTI quartile had a significantly greater risk of these chronic conditions than those in the lowest quartile. Furthermore, RCS analysis revealed a significant nonlinear relationship between the CTI and both stroke and osteoarthritis. These associations remained robust after adjustment for covariates and were consistent across sensitivity and subgroup analyses. These results support the potential of CTI as a novel biomarker for identifying high‐risk individuals, particularly those with metabolic dysfunction, and underscore its role in guiding early prevention strategies for chronic diseases.

The TyG index has emerged as a widely accepted surrogate marker for IR (Khan et al. 2018; Minh et al. 2021). It has been shown to be associated with the risk of hypertension (Ishida et al. 2025), cardiovascular disease (CVD) (Sun et al. 2025), heart disease (Chen et al. 2025), Type 2 diabetes (Luo et al. 2025), and nonalcoholic fatty liver disease (Cao et al. 2025). CRP, a commonly used marker of inflammation, has also been linked to the risk of stroke and cognitive impairment (Kuo et al. 2005). A prospective study from Spain indicated that higher CRP levels were associated with an increased risk of developing Type 2 diabetes over a 5‐year period (Rubio‐Martín et al. 2013). The CTI is a novel biomarker that combines the TyG index and CRP. Initially, the CTI was developed to predict cancer mortality in the general population (Zhao 2023). Later, a prospective study involving 8679 individuals demonstrated a significant association between elevated CTI levels and increased CVD incidence (Sun et al. 2025). Research on heart failure patients has also highlighted a nonlinear relationship between the CTI and its incidence (Cheng et al. 2025). Additionally, some studies have shown a significant association between higher CTI levels and CVD mortality (Sun et al. 2025). However, our study did not find a significant association between CTI and heart disease, suggesting that the impact of the CTI on heart disease might vary depending on disease subtype or pathological state. Future research should explore the relationships between the CTI and various subtypes of CVDs in more detail. Additionally, previous studies have shown a positive linear relationship between CTI levels and stroke incidence in middle‐aged and elderly populations (Tang et al. 2024). However, in our study, we found a nonlinear relationship between the CTI and stroke risk. Specifically, when the CTI was less than 5.173, each 1‐unit increase in the CTI was associated with a 70.9% increased risk of stroke. However, when the CTI exceeded 5.173, the stroke risk plateaued. This nonlinear relationship may be attributed to the close association between elevated CTI and the onset of conditions such as arteriosclerosis, glucose abnormalities, and CVDs. Moreover, we observed that as the CTI increased, the incidence of hypertension, diabetes, and dyslipidemia also increased. Therefore, once the CTI reaches a certain threshold, the cumulative effect of these comorbidities may become the primary driver of stroke risk.

In our study, we observed a positive association between elevated CTI levels and the risk of dyslipidemia, which is consistent with previous research showing a positive correlation between hs‐CRP and dyslipidemia (Bains et al. 2024). This relationship may be explained by the fact that inflammation increases the release of fatty acids, leading to elevated TG and LDL‐C levels (Feingold and Grunfeld 2000). Additionally, studies have shown a significant correlation between the TyG index and dyslipidemia, particularly with elevated TG and LDL‐C levels (Kim et al. 2023). This index reflects IR and lipid metabolism disturbances, which are often linked to dyslipidemia (Sharafi et al. 2023). Our study also revealed a positive association between CTI and diabetes risk, especially in female participants, most of whom were aged 45 years and older, with many in the peri‐ or postmenopausal phase. Estrogen has been shown to have a protective effect against IR and diabetes (De Paoli et al. 2021), and as estrogen levels decrease during menopause, the risk of IR increases. Therefore, monitoring CTI levels, particularly demographics, is essential for the early detection and prevention of diabetes. Moreover, we are the first to report a significant association between elevated CTI and the risk of osteoarthritis, a common chronic degenerative joint disease (Li et al. 2024). The pathogenesis of osteoarthritis has evolved from a mechanical wear‐and‐tear model to a multifactorial process driven by metabolic inflammation (Sampath et al. 2023; Shumnalieva et al. 2023), with IR and systemic low‐grade inflammation playing key roles in its onset and progression (Mocanu et al. 2024; Huang et al. 2025). Our study further identifies a nonlinear relationship between elevated CTI and osteoarthritis risk. Specifically, when CTI levels are low, the risk of osteoarthritis increases significantly, particularly below the threshold of K = 4.311. This finding underscores the importance of early intervention and management of CTI in individuals with low CTI levels, as the risk of osteoarthritis is notably higher at these levels. As CTI levels surpass this threshold, the risk increase plateaus, indicating that CTI monitoring may be crucial for the early prevention of osteoarthritis.

Although the exact mechanisms underlying the relationship between the CTI and disease risk are not fully understood, several potential mechanisms may explain this association. First, CTI serves as a marker of IR, which is a key pathological mechanism in metabolic diseases such as diabetes, hypertension, and dyslipidemia (Zhao et al. 2023). Elevated CTI reflects increased IR, leading to metabolic disturbances that impair glucose and lipid metabolism, thereby increasing the risk of these diseases (Sun et al. 2025; Chen et al. 2025; Chen et al. 2025; Ma et al. 2025). Second, the CTI reflects chronic low‐grade inflammation, which damages vascular endothelial function and promotes the development of hypertension and stroke, among other CVDs (Sun et al. 2025; Hage et al. 2024). Inflammation also affects insulin sensitivity in adipocytes and muscle cells, further exacerbating fat deposition and dyslipidemia and increasing the risk of nonalcoholic fatty liver disease (Monteiro and Azevedo 2010; Blaszczak et al. 2020; Hall et al. 2015; Mir et al. 2022; Wiering and Tacke 2023). Additionally, chronic low‐grade inflammation can lead to cartilage degradation and the progression of osteoarthritis (Karaman et al. 2025; Ghafari et al. 2025). Finally, elevated CTI is linked to increased oxidative stress (Rafiei et al. 2021; Yang et al. 2024), which damages the endothelium and promotes vasoconstriction, vascular sclerosis, and atherosclerosis, increasing the risk of hypertension, stroke, and CVDs (Boussekine et al. 2021; Griendling et al. 2021; Menon et al. 2020).

This study is innovative in its use of large‐scale, nationally representative longitudinal data to investigate the association between the CTI and the risk of 14 chronic diseases. Our results suggest that the CTI may serve as a novel and cost‐effective biomarker for predicting multiple chronic diseases. Furthermore, subgroup analyses revealed consistent findings across various population characteristics, offering valuable insights for clinical practice. However, several limitations must be acknowledged. First, the diagnosis of chronic diseases and covariates relied on self‐reported data, which may introduce recall bias. Nonetheless, previous validation studies have confirmed the reliability of these self‐reported diagnoses, supporting the credibility of the data (Yuan et al. 2015). Second, a large proportion of participants were excluded due to missing CTI data. Although the excluded and included groups were similar across many key clinical variables, differences in certain demographic and laboratory parameters may have introduced selection bias. This potential bias could limit the external validity of our findings. Third, while we controlled for a range of confounding factors, residual confounding cannot be entirely ruled out. Finally, as the study population is primarily from China, the findings may not be fully generalizable to other populations. Further validation in diverse populations is warranted.

## 5 Conclusion

The CTI exhibited a strong longitudinal association with the risk of chronic diseases, particularly diabetes, dyslipidemia, and stroke, in this national prospective longitudinal study. These findings suggest that the CTI could serve as an effective biomarker for the early identification of chronic disease risk.

## Author Contributions

**Huang Luwen**: conceptualization, methodology, data curation, formal analysis, and writing – original draft. **Mei Lijun**: conceptualization, methodology, formal analysis, and writing – original draft. **Li Linlin**: methodology, formal analysis, visualization, writing – review and editing. **Yu Ming**: conceptualization, methodology, supervision, writing – review and editing.

## Funding

The authors have nothing to report.

## Ethics Statement

The CHARLS study was approved by the Biomedical Ethics Review Committee of Peking University (IRB00001052–11015), and written informed consent was obtained from all participants.

## Conflicts of Interest

The authors declare no conflicts of interests.

## Supporting information



Table S1 Baseline characteristics of participants included in the analysis and excluded due to missing CTI data.
Table S2 Baseline characteristics of participants included in the analysis and excluded due to missing CTI data
Table S3. Logistic regression analysis of the association between CTI quartiles and new‐onset chronic diseases in CHARLS participants.
Table S4. Cox regression analysis of the association between CTI quartiles and new‐onset chronic diseases in CHARLS participants.
Table S5. Association between CTI and new‐onset chronic diseases in CHARLS participants after excluding those who experienced outcome events during wave 2.

## Data Availability

The data that support the findings of this study are openly available at the CHARLS home website (http://charls.pku.edu.cn/en).
