# Supplementary material for: The Role of the C‐Reactive Protein–Triglyceride Glucose Index in Predicting New‐Onset Chronic Diseases: Evidence From a Longitudinal Cohort Study
Source: Brain Behav. 2026 Feb 28;16(3):e71299. doi: 10.1002/brb3.71299 (PMC12949722; doi:10.1002/brb3.71299)
Supplement: Supplementary file 1 — Table S1 Baseline characteristics of participants included in the analysis and excluded due to missing CTI data. Table S2 Baseline characteristics of participants included in the analysis and excluded due to missing CTI data Table S3. Logistic regression analysis of the association between CTI quartiles and new‐onset chronic diseases in CHARLS participants. Table S4. Cox regression analysis of the association between CTI quartiles and new‐onset chronic diseases in CHARLS participants. Table S5. Association between CTI and new‐onset chronic diseases in CHARLS participants after excluding those who experienced outcome events during wave 2. [file BRB3-16-e71299-s001.docx]

| Table S1 Baseline characteristics of participants included in the analysis and excluded due to missing CTI data | | | | |
| --- | --- | --- | --- | --- |
| **Variables** | **Total** | **Excluded participants missing CTI (n = 6,072)** | **Included participants with CTI (n = 11,636)** | ***P*** |
| Age, years | 58.87 ± 10.17 | 58.63 ± 10.82 | 59.00 ± 9.82 | 0.021 |
| Sex (Female), n (%) | 8479 (47.89) | 3073 (50.63) | 5406 (46.46) | < 0.001 |
| Marital status (Married), n (%) | 15417 (87.22) | 5199 (85.98) | 10218 (87.87) | < 0.001 |
| Residence (Urban), n (%) | 3969 (22.47) | 1774 (29.37) | 2195 (18.89) | < 0.001 |
| Education level, n (%) |  |  |  | < 0.001 |
| Elementary school or below | 11744 (66.32) | 3716 (61.58) | 5535 (69.14) |  |
| Middle school | 3669 (20.79) | 1322 (21.91) | 2347 (20.21) |  |
| High school or above | 2233 (12.65) | 996 (16.51) | 1237 (10.65) |  |
| Drinking status, no (%) |  |  |  | 0.739 |
| Never | 10333 (58.90) | 3494 (58.59) | 6839 (59.06) |  |
| Former | 1443 ( 8.23) | 486 (8.15) | 957 (8.26) |  |
| Now | 5767 (32.87) | 1983 (33.26) | 3784 (32.68) |  |
| Smoking status, no (%) |  |  |  | 0.169 |
| Never | 10619 (62.81) | 3537 (63.76) | 7082 (62.34) |  |
| Former | 1417 ( 8.38) | 444 (8) | 973 (8.57) |  |
| Now | 4871 (28.81) | 1566 (28.23) | 3305 (29.09) |  |
| BMI (kg/m2) | 23.10 (20.79, 25.74) | 22.95 (20.64, 25.52) | 23.15 (20.85, 25.82) | 0.002 |
| **Laboratory parameters** |  |  |  |  |
| TC (mg/dl) | 192.97 ± 38.89 | 174.48 ± 41.74 | 193.00 ± 38.88 | 0.038 |
| LDL-C (mg/dl) | 115.99 ± 34.91 | 105.61 ± 33.68 | 116.02 ± 34.91 | 0.122 |
| HDL-C (mg/dl) | 50.84 ± 15.33 | 40.82 ± 8.14 | 50.87 ± 15.33 | < 0.001 |
| BUN (mg/dl) | 15.75 ± 4.65 | 16.43 ± 4.77 | 15.75 ± 4.65 | 0.44 |
| UA (mg/dl) | 4.46 ± 1.27 | 4.64 ± 1.27 | 4.46 ± 1.27 | 0.466 |
| **Chronic diseases** |  |  |  |  |
| Hypertension, no (%) | 7654 (43.71) | 2375 (39.88) | 5279 (45.69) | < 0.001 |
| Dyslipidemia, no (%) | 1696 ( 9.85) | 530 (9.04) | 1166 (10.27) | 0.011 |
| Diabetes, no (%) | 2115 (12.13) | 344 (5.8) | 1771 (15.4) | < 0.001 |
| Heart disease, no (%) | 2130 (12.19) | 691 (11.63) | 1439 (12.48) | 0.104 |
| Stroke, no (%) | 486 ( 2.77) | 164 (2.75) | 322 (2.79) | 0.883 |
| Lung disease, no (%) | 1704 ( 9.74) | 573 (9.62) | 1131 (9.8) | 0.703 |
| Asthma, no (%) | 773 ( 4.42) | 257 (4.32) | 516 (4.47) | 0.644 |
| Liver disease, no (%) | 605 ( 3.47) | 201 (3.39) | 404 (3.51) | 0.67 |
| Kidney disease, no (%) | 979 ( 5.61) | 303 (5.1) | 676 (5.87) | 0.037 |
| Digestive disease, no (%) | 3780 (21.58) | 1182 (19.83) | 2598 (22.48) | < 0.001 |
| Psychiatric disease, no (%) | 258 ( 1.48) | 96 (1.61) | 162 (1.4) | 0.276 |
| Memory disease, no (%) | 333 ( 1.90) | 142 (2.38) | 191 (1.65) | < 0.001 |
| Osteoarthritis, n(%) | 5706 (32.56) | 1716 (28.78) | 3990 (34.51) | < 0.001 |
| Cancer, no (%) | 167 ( 0.96) | 57 (0.96) | 110 (0.95) | 0.981 |

BMI, body mass index; CRP, C-reactive protein; FBG, fasting blood glucose; TC, total cholesterol; TG, triglycerides; LDL-C, low-density lipoprotein cholesterol; HDL-C, high-density lipoprotein cholesterol; BUN, blood urea nitrogen; UA, uric acid.

| Table S2 Baseline characteristics of participants included in the analysis and excluded due to missing CTI data | | | | |
| --- | --- | --- | --- | --- |
| **Variables** | **Total** | **Excluded participants missing CTI**  **(n = 6,072)** | **Included participants with CTI (n = 11,636)** | ***P*** |
| Age, years | 58.87 ± 10.17 | 58.63 ± 10.82 | 59.00 ± 9.82 | 0.021 |
| Sex (Female), n (%) | 8479 (47.89) | 3073 (50.63) | 5406 (46.46) | < 0.001 |
| Marital status (Married), n (%) | 15417 (87.22) | 5199 (85.98) | 10218 (87.87) | < 0.001 |
| Residence (Urban), n (%) | 3969 (22.47) | 1774 (29.37) | 2195 (18.89) | < 0.001 |
| Education level, n (%) |  |  |  | < 0.001 |
| Elementary school or below | 11744 (66.32) | 3716 (61.58) | 5535 (69.14) |  |
| Middle school | 3669 (20.79) | 1322 (21.91) | 2347 (20.21) |  |
| High school or above | 2233 (12.65) | 996 (16.51) | 1237 (10.65) |  |
| Drinking status, no (%) |  |  |  | 0.739 |
| Never | 10333 (58.90) | 3494 (58.59) | 6839 (59.06) |  |
| Former | 1443 ( 8.23) | 486 (8.15) | 957 (8.26) |  |
| Now | 5767 (32.87) | 1983 (33.26) | 3784 (32.68) |  |
| Smoking status, no (%) |  |  |  | 0.169 |
| Never | 10619 (62.81) | 3537 (63.76) | 7082 (62.34) |  |
| Former | 1417 ( 8.38) | 444 (8) | 973 (8.57) |  |
| Now | 4871 (28.81) | 1566 (28.23) | 3305 (29.09) |  |
| BMI (kg/m^2^) | 23.10 (20.79, 25.74) | 22.95 (20.64, 25.52) | 23.15 (20.85, 25.82) | 0.002 |
| **Laboratory parameters** |  |  |  |  |
| TC (mg/dl) | 192.97 ± 38.89 | 174.48 ± 41.74 | 193.00 ± 38.88 | 0.038 |
| LDL-C (mg/dl) | 115.99 ± 34.91 | 105.61 ± 33.68 | 116.02 ± 34.91 | 0.122 |
| HDL-C (mg/dl) | 50.84 ± 15.33 | 40.82 ± 8.14 | 50.87 ± 15.33 | < 0.001 |
| BUN (mg/dl) | 15.75 ± 4.65 | 16.43 ± 4.77 | 15.75 ± 4.65 | 0.44 |
| UA (mg/dl) | 4.46 ± 1.27 | 4.64 ± 1.27 | 4.46 ± 1.27 | 0.466 |
| **Chronic diseases** |  |  |  |  |
| Hypertension, no (%) | 7654 (43.71) | 2375 (39.88) | 5279 (45.69) | < 0.001 |
| Dyslipidemia, no (%) | 1696 ( 9.85) | 530 (9.04) | 1166 (10.27) | 0.011 |
| Diabetes, no (%) | 2115 (12.13) | 344 (5.8) | 1771 (15.4) | < 0.001 |
| Heart disease, no (%) | 2130 (12.19) | 691 (11.63) | 1439 (12.48) | 0.104 |
| Stroke, no (%) | 486 ( 2.77) | 164 (2.75) | 322 (2.79) | 0.883 |
| Lung disease, no (%) | 1704 ( 9.74) | 573 (9.62) | 1131 (9.8) | 0.703 |
| Asthma, no (%) | 773 ( 4.42) | 257 (4.32) | 516 (4.47) | 0.644 |
| Liver disease, no (%) | 605 ( 3.47) | 201 (3.39) | 404 (3.51) | 0.67 |
| Kidney disease, no (%) | 979 ( 5.61) | 303 (5.1) | 676 (5.87) | 0.037 |
| Digestive disease, no (%) | 3780 (21.58) | 1182 (19.83) | 2598 (22.48) | < 0.001 |
| Psychiatric disease, no (%) | 258 ( 1.48) | 96 (1.61) | 162 (1.4) | 0.276 |
| Memory disease, no (%) | 333 ( 1.90) | 142 (2.38) | 191 (1.65) | < 0.001 |
| Osteoarthritis, n(%) | 5706 (32.56) | 1716 (28.78) | 3990 (34.51) | < 0.001 |
| Cancer, no (%) | 167 ( 0.96) | 57 (0.96) | 110 (0.95) | 0.981 |

| Table S3. Logistic regression analysis of the association between CTI quartiles and new-onset chronic diseases in CHARLS participants. | | | | | | | |
| --- | --- | --- | --- | --- | --- | --- | --- |
| Variables | Event, (n%) | Model 1 | | Model 2 | | Model 3 | |
|  |  | OR (95%CI) | P value | OR (95%CI) | P value | OR (95%CI) | P value |
| Hypertension |  |  |  |  |  |  |  |
| Q1 | 406 (33.5) | 1(Ref) |  | 1(Ref) |  | 1(Ref) |  |
| Q2 | 471 (38.9) | 1.262  (1.069, 1.49) | 0.006 | 1.184  (1, 1.402) | 0.05 | 1.157  (0.966, 1.386) | 0.112 |
| Q3 | 513 (42.4) | 1.457  (1.236, 1.719) | <0.001 | 1.32  (1.114, 1.565) | 0.001 | 1.298  (1.082, 1.558) | 0.005 |
| Q4 | 572 (47.2) | 1.775  (1.506, 2.092) | <0.001 | 1.585  (1.336, 1.88) | <0.001 | 1.596  (1.325, 1.923) | <0.001 |
| P for trend |  | 1.205  (1.144, 1.269) | <0.001 | 1.161  (1.1, 1.225) | <0.001 | 1.164  (1.097, 1.235) | <0.001 |
| Dyslipidemia |  |  |  |  |  |  |  |
| Q1 | 377 (18) | 1(Ref) |  | 1(Ref) |  | 1(Ref) |  |
| Q2 | 440 (21.1) | 1.219  (1.045, 1.421) | 0.012 | 1.252  (1.072, 1.461) | 0.005 | 1.188  (1.005, 1.405) | 0.044 |
| Q3 | 542 (25.9) | 1.615  (1.392, 1.874) | <0.001 | 1.64  (1.411, 1.907) | <0.001 | 1.413  (1.199, 1.666) | <0.001 |
| Q4 | 650 (31.1) | 2.14  (1.85, 2.476) | <0.001 | 2.184  (1.883, 2.533) | <0.001 | 1.744  (1.472, 2.067) | <0.001 |
| P for trend |  | 1.295  (1.237, 1.356) | <0.001 | 1.301  (1.241, 1.363) | <0.001 | 1.203  (1.14, 1.269) | <0.001 |
| Diabetes |  |  |  |  |  |  |  |
| Q1 | 182 (9.2) | 1(Ref) |  | 1(Ref) |  | 1(Ref) |  |
| Q2 | 226 (11.4) | 1.285  (1.045, 1.579) | 0.017 | 1.276  (1.037, 1.569) | 0.021 | 1.218  (0.973, 1.524) | 0.085 |
| Q3 | 305 (15.4) | 1.825  (1.501, 2.219) | <0.001 | 1.821  (1.496, 2.218) | <0.001 | 1.695  (1.369, 2.1) | <0.001 |
| Q4 | 409 (20.7) | 2.653  (2.199, 3.201) | <0.001 | 2.65  (2.192, 3.204) | <0.001 | 2.425  (1.963, 2.995) | <0.001 |
| P for trend |  | 1.396  (1.316, 1.48) | <0.001 | 1.396  (1.316, 1.482) | <0.001 | 1.357  (1.27, 1.451) | <0.001 |
| Heart disease |  |  |  |  |  |  |  |
| Q1 | 318 (15.6) | 1(Ref) |  | 1(Ref) |  | 1(Ref) |  |
| Q2 | 377 (18.5) | 1.235  (1.048, 1.456) | 0.012 | 1.186  (1.005, 1.4) | 0.044 | 1.108  (0.925, 1.327) | 0.266 |
| Q3 | 394 (19.3) | 1.311  (1.114, 1.543) | 0.001 | 1.216  (1.031, 1.435) | 0.02 | 1.148  (0.958, 1.374) | 0.134 |
| Q4 | 420 (20.6) | 1.45  (1.234, 1.703) | <0.001 | 1.335  (1.133, 1.573) | <0.001 | 1.216  (1.007, 1.469) | 0.043 |
| P for trend |  | 1.123  (1.067, 1.181) | <0.001 | 1.092  (1.037, 1.149) | <0.001 | 1.064  (1.002, 1.129) | 0.042 |
| Stroke |  |  |  |  |  |  |  |
| Q1 | 125 (5.5) | 1(Ref) |  | 1(Ref) |  | 1(Ref) |  |
| Q2 | 182 (8) | 1.509  (1.192, 1.91) | <0.001 | 1.449  (1.143, 1.836) | 0.002 | 1.4  (1.08, 1.815) | 0.011 |
| Q3 | 227 (10) | 1.93  (1.538, 2.421) | <0.001 | 1.82  (1.448, 2.288) | <0.001 | 1.658  (1.289, 2.132) | <0.001 |
| Q4 | 284 (12.6) | 2.524  (2.026, 3.143) | <0.001 | 2.361  (1.892, 2.947) | <0.001 | 2.053  (1.59, 2.651) | <0.001 |
| P for trend |  | 1.344  (1.257, 1.436) | <0.001 | 1.317  (1.231, 1.408) | <0.001 | 1.255  (1.16, 1.357) | <0.001 |
| Lung disease |  |  |  |  |  |  |  |
| Q1 | 281 (13.4) | 1(Ref) |  | 1(Ref) |  | 1(Ref) |  |
| Q2 | 317 (15.2) | 1.159  (0.974, 1.379) | 0.096 | 1.124  (0.943, 1.339) | 0.192 | 1.09  (0.897, 1.324) | 0.386 |
| Q3 | 322 (15.4) | 1.185  (0.997, 1.409) | 0.054 | 1.163  (0.976, 1.387) | 0.092 | 1.211  (0.994, 1.474) | 0.057 |
| Q4 | 334 (16) | 1.254  (1.056, 1.49) | 0.01 | 1.227  (1.03, 1.463) | 0.022 | 1.278  (1.041, 1.569) | 0.019 |
| P for trend |  | 1.072  (1.016, 1.131) | 0.012 | 1.066  (1.009, 1.127) | 0.023 | 1.088  (1.019, 1.161) | 0.012 |
| Asthma |  |  |  |  |  |  |  |
| Q1 | 91 (4.1) | 1(Ref) |  | 1(Ref) |  | 1(Ref) |  |
| Q2 | 95 (4.3) | 1.052  (0.784, 1.411) | 0.738 | 0.996  (0.741, 1.339) | 0.978 | 0.956  (0.693, 1.319) | 0.786 |
| Q3 | 113 (5.1) | 1.267  (0.955, 1.681) | 0.101 | 1.202  (0.904, 1.6) | 0.206 | 1.228  (0.9, 1.676) | 0.194 |
| Q4 | 109 (4.9) | 1.236  (0.929, 1.643) | 0.145 | 1.174  (0.88, 1.567) | 0.276 | 1.099  (0.792, 1.526) | 0.571 |
| P for trend |  | 1.085  (0.992, 1.186) | 0.073 | 1.069  (0.977, 1.171) | 0.148 | 1.056  (0.953, 1.171) | 0.298 |
| Liver disease |  |  |  |  |  |  |  |
| Q1 | 145 (6.5) | 1(Ref) |  | 1(Ref) |  | 1(Ref) |  |
| Q2 | 162 (7.2) | 1.134  (0.899, 1.43) | 0.289 | 1.144  (0.906, 1.445) | 0.258 | 1.123  (0.875, 1.441) | 0.364 |
| Q3 | 182 (8.1) | 1.292  (1.03, 1.62) | 0.027 | 1.306  (1.039, 1.641) | 0.022 | 1.304  (1.02, 1.667) | 0.034 |
| Q4 | 192 (8.6) | 1.389  (1.11, 1.738) | 0.004 | 1.408  (1.122, 1.767) | 0.003 | 1.307  (1.008, 1.658) | 0.044 |
| P for trend |  | 1.117  (1.041, 1.198) | 0.002 | 1.122  (1.044, 1.204) | 0.002 | 1.094  (1.01, 1.184) | 0.027 |
| Kidney disease |  |  |  |  |  |  |  |
| Q1 | 137 (6.3) | 1(Ref) |  | 1(Ref) |  | 1(Ref) |  |
| Q2 | 164 (7.5) | 1.22  (0.964, 1.544) | 0.097 | 1.197  (0.945, 1.516) | 0.137 | 1.149  (0.887, 1.488) | 0.293 |
| Q3 | 160 (7.3) | 1.191  (0.94, 1.509) | 0.147 | 1.169  (0.921, 1.485) | 0.2 | 1.167  (0.897, 1.517) | 0.251 |
| Q4 | 168 (7.7) | 1.278  (1.012, 1.616) | 0.04 | 1.248  (0.984, 1.584) | 0.068 | 1.071  (0.81, 1.415) | 0.631 |
| P for trend |  | 1.072  (0.997, 1.153) | 0.062 | 1.064  (0.988, 1.146) | 0.1 | 1.021  (0.936, 1.114) | 0.639 |
| Digestive disease |  |  |  |  |  |  |  |
| Q1 | 489 (27.3) | 1(Ref) |  | 1(Ref) |  | 1(Ref) |  |
| Q2 | 434 (24.2) | 0.861  (0.741, 1.001) | 0.052 | 0.889  (0.764, 1.035) | 0.129 | 0.884  (0.751, 1.04) | 0.137 |
| Q3 | 445 (24.8) | 0.89  (0.767, 1.034) | 0.129 | 0.921  (0.79, 1.072) | 0.288 | 0.934  (0.793, 1.101) | 0.419 |
| Q4 | 416 (23.2) | 0.827  (0.711, 0.963) | 0.014 | 0.864  (0.739, 1.01) | 0.067 | 0.832  (0.699, 1.009) | 0.054 |
| P for trend |  | 0.947  (0.903, 0.994) | 0.028 | 0.96  (0.914, 1.009) | 0.108 | 0.952  (0.901, 1.006) | 0.079 |
| Psychiatric disease |  |  |  |  |  |  |  |
| Q1 | 86 (3.8) | 1(Ref) |  | 1(Ref) |  | 1(Ref) |  |
| Q2 | 96 (4.2) | 1.151  (0.847, 1.563) | 0.369 | 1.172  (0.86, 1.596) | 0.315 | 1.152  (0.822, 1.615) | 0.41 |
| Q3 | 82 (3.6) | 0.958  (0.696, 1.319) | 0.792 | 0.958  (0.691, 1.327) | 0.796 | 0.852  (0.591, 1.229) | 0.392 |
| Q4 | 66 (2.9) | 0.787  (0.562, 1.102) | 0.164 | 0.787  (0.556, 1.114) | 0.177 | 0.732  (0.493, 1.086) | 0.121 |
| P for trend |  | 0.918  (0.829, 1.017) | 0.101 | 0.916  (0.824, 1.019) | 0.106 | 0.888  (0.786, 1.003) | 0.056 |
| Memory disease |  |  |  |  |  |  |  |
| Q1 | 142 (6.2) | 1(Ref) |  | 1(Ref) |  | 1(Ref) |  |
| Q2 | 165 (7.2) | 1.183  (0.938, 1.493) | 0.156 | 1.094  (0.865, 1.385) | 0.452 | 1.063  (0.837, 1.349) | 0.616 |
| Q3 | 183 (8) | 1.329  (1.058, 1.668) | 0.014 | 1.182  (0.937, 1.49) | 0.158 | 1.106  (0.873, 1.401) | 0.406 |
| Q4 | 174 (7.6) | 1.276  (1.014, 1.605) | 0.038 | 1.129  (0.893, 1.427) | 0.31 | 0.977  (0.763, 1.251) | 0.855 |
| P for trend |  | 1.086  (1.011, 1.165) | 0.023 | 1.043  (0.97, 1.122) | 0.254 | 0.996  (0.922, 1.075) | 0.913 |
| Osteoarthritis |  |  |  |  |  |  |  |
| Q1 | 452 (29.7) | 1(Ref) |  | 1(Ref) |  | 1(Ref) |  |
| Q2 | 483 (31.8) | 1.115  (0.955, 1.301) | 0.17 | 1.132  (0.967, 1.324) | 0.123 | 1.163  (0.992, 1.363) | 0.063 |
| Q3 | 479 (31.5) | 1.11  (0.95, 1.296) | 0.188 | 1.146  (0.978, 1.342) | 0.091 | 1.203  (1.023, 1.413) | 0.025 |
| Q4 | 460 (30.2) | 1.061  (0.908, 1.241) | 0.454 | 1.102  (0.939, 1.292) | 0.235 | 1.186  (1.003, 1.402) | 0.046 |
| P for trend |  | 1.018  (0.969, 1.069) | 0.485 | 1.031  (0.98, 1.084) | 0.24 | 1.057  (1.002, 1.114) | 0.041 |
| Cancer |  |  |  |  |  |  |  |
| Q1 | 75 (3.3) | 1(Ref) |  | 1(Ref) |  | 1(Ref) |  |
| Q2 | 64 (2.8) | 0.847  (0.598, 1.201) | 0.351 | 0.85  (0.599, 1.206) | 0.362 | 0.867  (0.61, 1.234) | 0.43 |
| Q3 | 58 (2.5) | 0.761  (0.532, 1.09) | 0.136 | 0.755  (0.525, 1.085) | 0.128 | 0.755  (0.523, 1.091) | 0.134 |
| Q4 | 83 (3.6) | 1.053  (0.755, 1.467) | 0.762 | 1.047  (0.748, 1.466) | 0.79 | 1.009  (0.707, 1.44) | 0.959 |
| P for trend |  | 1.007  (0.901, 1.125) | 0.903 | 1.005  (0.898, 1.124) | 0.934 | 0.989  (0.879, 1.112) | 0.854 |

Model 1: Crude model;

Model 2: Adjusted for age, sex, education level, drinking status, smoking status, and BMI;

Model 3: Adjusted for age, sex, education level, drinking status, smoking status, BMI, LDL-C

and the history of 14 chronic diseases at baseline (excluding the chronic disease being studied in each cohort). In the dyslipidemia, TC was additionally included. In the kidney disease, UA, creatinine, and BUN were further adjusted.

| Table S4. Cox regression analysis of the association between CTI quartiles and new-onset chronic diseases in CHARLS participants. | | | | | | | |
| --- | --- | --- | --- | --- | --- | --- | --- |
| Variables | Event, (n%) | Model 1 | | Model 2 | | Model 3 | |
|  |  | HR (95%CI) | P value | HR (95%CI) | P value | HR (95%CI) | P value |
| Hypertension |  |  |  |  |  |  |  |
| Q1 | 406 (33.5) | 1(Ref) |  | 1(Ref) |  | 1(Ref) |  |
| Q2 | 471 (38.9) | 1.218  (1.067, 1.391) | 0.004 | 1.173  (1.027, 1.34) | 0.019 | 1.152  (1.008, 1.316) | 0.038 |
| Q3 | 513 (42.4) | 1.362  (1.196, 1.552) | <0.001 | 1.296  (1.137, 1.477) | <0.001 | 1.262  (1.106, 1.44) | <0.001 |
| Q4 | 572 (47.2) | 1.673  (1.473, 1.9) | <0.001 | 1.601  (1.408, 1.82) | <0.001 | 1.548  (1.358, 1.765) | <0.001 |
| P for trend |  | 1.18  (1.134, 1.228) | <0.001 | 1.164  (1.118, 1.213) | <0.001 | 1.152  (1.105, 1.201) | <0.001 |
| Dyslipidemia |  |  |  |  |  |  |  |
| Q1 | 377 (18) | 1(Ref) |  | 1(Ref) |  | 1(Ref) |  |
| Q2 | 440 (21.1) | 1.232  (1.074, 1.414) | 0.003 | 1.23  (1.072, 1.412) | 0.003 | 1.139  (0.991, 1.308) | 0.066 |
| Q3 | 542 (25.9) | 1.611  (1.413, 1.838) | <0.001 | 1.577  (1.382, 1.801) | <0.001 | 1.373  (1.201, 1.57) | <0.001 |
| Q4 | 650 (31.1) | 2.135  (1.88, 2.424) | <0.001 | 2.086  (1.835, 2.371) | <0.001 | 1.741  (1.524, 1.988) | <0.001 |
| P for trend |  | 1.293  (1.242, 1.346) | <0.001 | 1.282  (1.231, 1.334) | <0.001 | 1.208  (1.158, 1.259) | <0.001 |
| Diabetes |  |  |  |  |  |  |  |
| Q1 | 182 (9.2) | 1(Ref) |  | 1(Ref) |  | 1(Ref) |  |
| Q2 | 226 (11.4) | 1.286  (1.058, 1.564) | 0.011 | 1.26  (1.036, 1.532) | 0.021 | 1.203  (0.988, 1.464) | 0.065 |
| Q3 | 305 (15.4) | 1.808  (1.504, 2.172) | <0.001 | 1.773  (1.474, 2.132) | <0.001 | 1.618  (1.343, 1.95) | <0.001 |
| Q4 | 409 (20.7) | 2.603  (2.186, 3.1) | <0.001 | 2.543  (2.132, 3.033) | <0.001 | 2.206  (1.843, 2.641) | <0.001 |
| P for trend |  | 1.387  (1.314, 1.464) | <0.001 | 1.379  (1.305, 1.456) | <0.001 | 1.315  (1.243, 1.391) | <0.001 |
| Heart disease |  |  |  |  |  |  |  |
| Q1 | 318 (15.6) | 1(Ref) |  | 1(Ref) |  | 1(Ref) |  |
| Q2 | 377 (18.5) | 1.239  (1.067, 1.438) | 0.005 | 1.167  (1.004, 1.355) | 0.044 | 1.134  (0.975, 1.318) | 0.103 |
| Q3 | 394 (19.3) | 1.334  (1.15, 1.546) | <0.001 | 1.217  (1.049, 1.413) | 0.010 | 1.134  (0.975, 1.318) | 0.103 |
| Q4 | 420 (20.6) | 1.489  (1.287, 1.722) | <0.001 | 1.353  (1.168, 1.567) | <0.001 | 1.171  (1.004, 1.365) | 0.045 |
| P for trend |  | 1.133  (1.083, 1.185) | <0.001 | 1.098  (1.049, 1.15) | <0.001 | 1.047  (0.998, 1.098) | 0.063 |
| Stroke |  |  |  |  |  |  |  |
| Q1 | 125 (5.5) | 1(Ref) |  | 1(Ref) |  | 1(Ref) |  |
| Q2 | 182 (8) | 1.529  (1.217, 1.919) | <0.001 | 1.442  (1.148, 1.812) | 0.002 | 1.334  (1.06, 1.678) | 0.014 |
| Q3 | 227 (10) | 1.971  (1.584, 2.452) | <0.001 | 1.824  (1.464, 2.271) | <0.001 | 1.588  (1.272, 1.984) | <0.001 |
| Q4 | 284 (12.6) | 2.625  (2.127, 3.24) | <0.001 | 2.411  (1.95, 2.981) | <0.001 | 1.926  (1.544, 2.402) | <0.001 |
| P for trend |  | 1.36  (1.277, 1.449) | <0.001 | 1.327  (1.244, 1.414) | <0.001 | 1.233  (1.152, 1.318) | <0.001 |
| Lung disease |  |  |  |  |  |  |  |
| Q1 | 281 (13.4) | 1(Ref) |  | 1(Ref) |  | 1(Ref) |  |
| Q2 | 317 (15.2) | 1.163  (0.99, 1.365) | 0.066 | 1.114  (0.948, 1.309) | 0.190 | 1.101  (0.936, 1.294) | 0.246 |
| Q3 | 322 (15.4) | 1.212  (1.033, 1.423) | 0.018 | 1.167  (0.993, 1.372) | 0.061 | 1.168  (0.992, 1.376) | 0.062 |
| Q4 | 334 (16) | 1.311  (1.119, 1.537) | <0.001 | 1.258  (1.071, 1.477) | 0.005 | 1.26  (1.066, 1.489) | 0.007 |
| P for trend |  | 1.088  (1.036, 1.144) | <0.001 | 1.076  (1.023, 1.131) | 0.005 | 1.078  (1.023, 1.136) | 0.005 |
| Asthma |  |  |  |  |  |  |  |
| Q1 | 91 (4.1) | 1(Ref) |  | 1(Ref) |  | 1(Ref) |  |
| Q2 | 95 (4.3) | 1.08  (0.81, 1.44) | 0.599 | 1.009  (0.756, 1.346) | 0.953 | 0.964  (0.72, 1.289) | 0.803 |
| Q3 | 113 (5.1) | 1.326  (1.006, 1.747) | 0.045 | 1.236  (0.936, 1.633) | 0.135 | 1.211  (0.913, 1.606) | 0.183 |
| Q4 | 109 (4.9) | 1.33  (1.007, 1.757) | 0.044 | 1.24  (0.937, 1.643) | 0.133 | 1.163  (0.866, 1.562) | 0.315 |
| P for trend |  | 1.111  (1.019, 1.212) | 0.018 | 1.089  (0.997, 1.19) | 0.059 | 1.072  (0.977, 1.177) | 0.144 |
| Liver disease |  |  |  |  |  |  |  |
| Q1 | 145 (6.5) | 1(Ref) |  | 1(Ref) |  | 1(Ref) |  |
| Q2 | 162 (7.2) | 1.151  (0.92, 1.44) | 0.219 | 1.137  (0.908, 1.423) | 0.264 | 1.113  (0.888, 1.396) | 0.352 |
| Q3 | 182 (8.1) | 1.327  (1.067, 1.65) | 0.011 | 1.301  (1.044, 1.621) | 0.019 | 1.259  (1.007, 1.574) | 0.043 |
| Q4 | 192 (8.6) | 1.459  (1.176, 1.81) | <0.001 | 1.433  (1.153, 1.783) | 0.001 | 1.261  (1.002, 1.588) | 0.048 |
| P for trend |  | 1.135  (1.061, 1.214) | <0.001 | 1.129  (1.054, 1.209) | <0.001 | 1.084  (1.009, 1.165) | 0.028 |
| Kidney disease |  |  |  |  |  |  |  |
| Q1 | 137 (6.3) | 1(Ref) |  | 1(Ref) |  | 1(Ref) |  |
| Q2 | 164 (7.5) | 1.247  (0.994, 1.565) | 0.057 | 1.202  (0.957, 1.51) | 0.113 | 1.181  (0.939, 1.486) | 0.154 |
| Q3 | 160 (7.3) | 1.226  (0.976, 1.541) | 0.080 | 1.186  (0.942, 1.494) | 0.147 | 1.112  (0.879, 1.406) | 0.377 |
| Q4 | 168 (7.7) | 1.349  (1.077, 1.691) | 0.009 | 1.301  (1.033, 1.638) | 0.025 | 1.149  (0.9, 1.467) | 0.266 |
| P for trend |  | 1.09  (1.016, 1.169) | 0.016 | 1.079  (1.004, 1.159) | 0.039 | 1.035  (0.958, 1.117) | 0.385 |
| Digestive disease |  |  |  |  |  |  |  |
| Q1 | 489 (27.3) | 1(Ref) |  | 1(Ref) |  | 1(Ref) |  |
| Q2 | 434 (24.2) | 0.91  (0.8, 1.036) | 0.153 | 0.918  (0.806, 1.045) | 0.196 | 0.902  (0.791, 1.028) | 0.123 |
| Q3 | 445 (24.8) | 0.945  (0.831, 1.074) | 0.385 | 0.955  (0.837, 1.089) | 0.490 | 0.942  (0.825, 1.075) | 0.376 |
| Q4 | 416 (23.2) | 0.91  (0.799, 1.038) | 0.159 | 0.928  (0.809, 1.063) | 0.280 | 0.903  (0.784, 1.04) | 0.157 |
| P for trend |  | 0.975  (0.935, 1.017) | 0.236 | 0.981  (0.939, 1.025) | 0.387 | 0.973  (0.93, 1.018) | 0.241 |
| Psychiatric disease |  |  |  |  |  |  |  |
| Q1 | 86 (3.8) | 1(Ref) |  | 1(Ref) |  | 1(Ref) |  |
| Q2 | 96 (4.2) | 1.15  (0.86, 1.539) | 0.346 | 1.149  (0.856, 1.541) | 0.355 | 1.105  (0.822, 1.484) | 0.509 |
| Q3 | 82 (3.6) | 1.005  (0.742, 1.36) | 0.976 | 0.992  (0.727, 1.354) | 0.961 | 0.952  (0.696, 1.304) | 0.761 |
| Q4 | 66 (2.9) | 0.841  (0.61, 1.159) | 0.291 | 0.839  (0.599, 1.175) | 0.307 | 0.716  (0.503, 1.019) | 0.064 |
| P for trend |  | 0.941  (0.854, 1.037) | 0.223 | 0.939  (0.847, 1.041) | 0.233 | 0.899  (0.808, 1.001) | 0.053 |
| Memory disease |  |  |  |  |  |  |  |
| Q1 | 142 (6.2) | 1(Ref) |  | 1(Ref) |  | 1(Ref) |  |
| Q2 | 165 (7.2) | 1.212  (0.969, 1.517) | 0.093 | 1.119  (0.894, 1.402) | 0.327 | 1.094  (0.872, 1.372) | 0.437 |
| Q3 | 183 (8) | 1.385  (1.112, 1.724) | 0.004 | 1.237  (0.992, 1.543) | 0.059 | 1.153  (0.921, 1.442) | 0.214 |
| Q4 | 174 (7.6) | 1.374  (1.101, 1.716) | 0.005 | 1.222  (0.977, 1.528) | 0.079 | 1.039  (0.822, 1.314) | 0.747 |
| P for trend |  | 1.112  (1.039, 1.19) | 0.002 | 1.071  (0.999, 1.148) | 0.053 | 1.016  (0.944, 1.092) | 0.678 |
| Osteoarthritis |  |  |  |  |  |  |  |
| Q1 | 452 (29.7) | 1(Ref) |  | 1(Ref) |  | 1(Ref) |  |
| Q2 | 483 (31.8) | 1.129  (0.993, 1.284) | 0.063 | 1.122  (0.987, 1.276) | 0.079 | 1.109  (0.967, 1.273) | 0.140 |
| Q3 | 479 (31.5) | 1.144  (1.006, 1.301) | 0.040 | 1.144  (1.005, 1.303) | 0.042 | 1.197  (1.042, 1.375) | 0.011 |
| Q4 | 460 (30.2) | 1.129  (0.991, 1.285) | 0.068 | 1.13  (0.991, 1.289) | 0.068 | 1.169  (1.01, 1.353) | 0.037 |
| P for trend |  | 1.038  (0.997, 1.081) | 0.070 | 1.039  (0.997, 1.083) | 0.067 | 1.057  (1.01, 1.106) | 0.018 |
| Cancer |  |  |  |  |  |  |  |
| Q1 | 75 (3.3) | 1(Ref) |  | 1(Ref) |  | 1(Ref) |  |
| Q2 | 64 (2.8) | 0.881  (0.631, 1.23) | 0.457 | 0.864  (0.618, 1.207) | 0.391 | 0.878  (0.611, 1.263) | 0.484 |
| Q3 | 58 (2.5) | 0.81  (0.575, 1.142) | 0.229 | 0.779  (0.552, 1.101) | 0.157 | 0.857  (0.593, 1.238) | 0.410 |
| Q4 | 83 (3.6) | 1.224  (0.896, 1.673) | 0.205 | 1.183  (0.863, 1.622) | 0.298 | 1.131  (0.789, 1.622) | 0.502 |
| P for trend |  | 1.059  (0.954, 1.177) | 0.283 | 1.047  (0.941, 1.165) | 0.396 | 1.036  (0.92, 1.167) | 0.562 |

Model 1: Crude model;

Model 2: Adjusted for age, sex, education level, drinking status, smoking status, and BMI;

Model 3: Adjusted for age, sex, education level, drinking status, smoking status, BMI, LDL-C

and the history of 14 chronic diseases at baseline (excluding the chronic disease being studied in each cohort). In the dyslipidemia, TC was additionally included. In the kidney disease, UA, creatinine, and BUN were further adjusted.

| Table S5. Association between CTI and new-onset chronic diseases in CHARLS participants after excluding those who experienced outcome events during wave 2 | | | | | | | |
| --- | --- | --- | --- | --- | --- | --- | --- |
| Variables | Event, (n%) | Model 1 | | Model 2 | | Model 3 | |
|  |  | HR (95%CI) | P value | HR (95%CI) | P value | HR (95%CI) | P value |
| Hypertension | 1031 (26.3) | 1.428  (1.287, 1.586) | **<0.001** | 1.405  (1.264, 1.561) | **<0.001** | 1.357  (1.205, 1.529) | **<0.001** |
| Dyslipidemia | 1721 (21.3) | 1.613  (1.492, 1.744) | **<0.001** | 1.592  (1.471, 1.724) | **<0.001** | 1.465  (1.338, 1.603) | **<0.001** |
| Diabetes | 1038 (13.3) | 1.873  (1.685, 2.082) | **<0.001** | 1.857  (1.668, 2.067) | **<0.001** | 1.754  (1.556, 1.976) | **<0.001** |
| Heart disease | 1320 (16.5) | 1.2  (1.096, 1.314) | <0.001 | 1.133  (1.032, 1.243) | 0.008 | 1.003  (0.902, 1.116) | 0.957 |
| Stroke | 767 (8.5) | 1.699  (1.518, 1.902) | **<0.001** | 1.624  (1.448, 1.821) | **<0.001** | 1.445  (1.275, 1.638) | **<0.001** |
| Lung disease | 1146 (13.9) | 1.113  (1.009, 1.228) | 0.033 | 1.087  (0.983, 1.201) | 0.105 | 1.12  (0.996, 1.26) | 0.058 |
| Asthma | 382 (4.3) | 1.239  (1.048, 1.465) | 0.012 | 1.202  (1.014, 1.426) | 0.034 | 1.113  (0.915, 1.353) | 0.283 |
| Liver disease | 601 (6.8) | 1.278  (1.118, 1.459) | **<0.001** | 1.266  (1.106, 1.448) | **<0.001** | 1.171  (1.006, 1.363) | **0.041** |
| Kidney disease | 510 (5.9) | 1.235  (1.068, 1.428) | 0.004 | 1.218  (1.048, 1.416) | 0.010 | 1.156  (0.972, 1.376) | 0.102 |
| Digestive disease | 1779 (24.8) | 0.976  (0.9, 1.057) | 0.547 | 0.99  (0.91, 1.077) | 0.820 | 0.979  (0.893, 1.074) | 0.660 |
| Psychiatric disease | 309 (3.4) | 0.937  (0.771, 1.137) | 0.508 | 0.933  (0.759, 1.147) | 0.512 | 0.854  (0.688, 1.061) | 0.155 |
| Memory disease | 613 (6.7) | 1.207  (1.057, 1.378) | 0.006 | 1.128  (0.984, 1.292) | 0.083 | 1.023  (0.884, 1.182) | 0.763 |
| Osteoarthritis | 1675 (28.5) | 1.069  (0.986, 1.16) | 0.106 | 1.072  (0.987, 1.165) | 0.099 | 1.104  (1.005, 1.213) | **0.040** |
| Cancer | 258 (2.8) | 1.083  (0.879, 1.334) | 0.454 | 1.063  (0.861, 1.313) | 0.571 | 1.038  (0.817, 1.32) | 0.758 |

Model 1: Crude model;

Model 2: Adjusted for age, sex, education level, drinking status, smoking status, and BMI;

Model 3: Adjusted for age, sex, education level, drinking status, smoking status, BMI, LDL-C

and the history of 14 chronic diseases at baseline (excluding the chronic disease being studied in each cohort). In the dyslipidemia, TC was additionally included. In the kidney disease, UA, creatinine, and BUN were further adjusted.
